# Transition and Group IIB Metal Complexes With “Active Aldehyde” Derivatives of Thiamine

**DOI:** 10.1155/MBD.1994.221

**Published:** 1994

**Authors:** Maria Louloudi, Nick Hadjiliadis, Jean Pierre Laussac, Robert Bau

**Affiliations:** 1 Technological Institution of Athens Department of Chemistry, Physics and Materials Technology Agiou Spyridonos and Pallikaridi Egaleo Athens Greece; 2 University of Ioannina Department of Chemistry Laboratory of Inorganic and General Chemistry Ioannina 45-110 Greece; 3 Laboratoire de Chimie de Coordination du CNRS 205 Route de Narbonne Toulouse Cedex F-31077 France; 4 University of Southern California Department of Chemistry California Los Angeles 90089-1062 USA

## Abstract

The Zn^2+^, Cd^2+^, Hg^2+^, Co^2+^ and Ni^2+^ ions produce zwitterionic type complexes with the ligands (L), 2-(α-hydroxy-benzyl)thiamine=HBT and 2-(α-hydroxy-cyclohexyl-methyl)thiamine = HCMT, of the type MLCl_3_. The ligands are in the S conformation, the metals are bound to N_1_, of the pyrimidine moiety of thiamine and the complexes have a trigonally distorted tetrahedral structure, as the crystal structure of the complex Zn(HCMT)Cl_3_ (orthorombic, a=14.4 b=14.1 c=17.4 β=105.6^O^ V=3392A^3^ R=13.8%), the one and two dimensional ^1^H nmr spectra of the Zn^2+^, Cd^2+^ and Hg^2+^ complexes and the electronic spectra of the Co^2+^ and Ni^2+^ complexes show. A brief review of the previous techniques (structure of the Hg(HBT)Cl_3_ complex, IR-Raman spectra, ^13^C nmr in solution and solid state etc) used to characterize these complexes, is also given here and the proper conclusions drawn.


### TRANSITION AND GROUP IIB METAL COMPLEXES WITH "ACTIVE ALDEHYDE" DERIVATIVES OF THIAMINE

Maria Louloudi[1], Nick Hadjiliadis*[1,2], Jean Pierre Laussac[3] and Robert Bau[4]

[1] Technological Institution of Athens, Department of Chemistry, Physics and Materials Technology, Agiou Spyridonos and Pallikaridi, Egaleo, Athens, Greece
[2] University of Ioannina, Department of Chemistry, Laboratory of Inorganic and General Chemistry, Ioannina 45-110, Greece
[3] Laboratoire de Chimie de Coordination du CNRS, 205 Route de Narbonne, F-31077 Toulouse, Cedex, France
[4] University of Southern California, Department of Chemistry, Los Angeles, 90089-1062, California, USA

Abstract: The $Zn^{2+}$, $Cd^{2+}$, $Hg^{2+}$, $Co^{2+}$ and $Ni^{2+}$ ions produce zwitterionic type complexes with the ligands (L), 2-(α-hydroxy-benzyl)thiamine=HBT and 2-(α-hydroxy-cyclohexyl-methyl)thiamine = HCMT, of the type $MLCl_3$. The ligands are in the S conformation, the metals are bound to $N_{1'}$ of the pyrimidine moiety of thiamine and the complexes have a trigonally distorted tetrahedral structure, as the crystal structure of the complex $Zn(HCMT)Cl_3$ (orthorombic, a=14.4 b=14.1 c=17.4 β=105.6$^o$ V=3392A$^3$ R=13.8%), the one and two dimensional $^1H$ nmr spectra of the $Zn^{2+}$, $Cd^{2+}$ and $Hg^{2+}$ complexes and the electronic spectra of the $Co^{2+}$ and $Ni^{2+}$ complexes show. A brief review of the previous techniques (structure of the $Hg(HBT)Cl_3$ complex, IR-Raman spectra, $^{13}C$ nmr in solution and solid state etc) used to characterize these complexes, is also given here and the proper conclusions drawn.

Introduction: The pyrophosphate ester of thiamine (vitamin $B_1$) is the coenzyme of many enzymes like carboxylase, transketolase etc, catalyzing the decarboxylation of α-ketoacids or the formation of α-ketols [1]. $Mg^{2+}$ ions are required in vivo for its action, but in vitro other bivalent metal ions like $Co^{2+}$, $Zn^{2+}$, $Ni^{2+}$ etc are also active as well. The enzymatic mechanism of action of thiamine enzymes requires the formation of the so called "active aldehyde" intermediates (I) which were isolated [1,2]. However the role played by the bivalent metal ions is not as yet quite clear. Attempts to prepare metal complexes of thiamine with

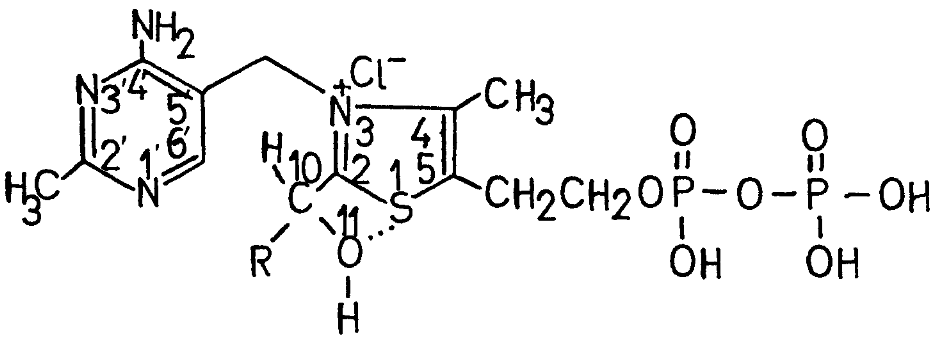

(I)

bivalent metals were resulted for many years to the formation of ionic salts of the type $[L]^{2+}[MX_4]^{2-}$, $[L]^{2+}([MX_3]^-)_2$, $([L]^+)_2[MX_4]^{2-}$, without a direct metal-ligand bonding, due to the net positive charge on thiamine and to the easy protonation of the $N_{1'}$ site of pyrimidine ($pK_a$~5) [3]. Despite this difficulty, a few metal complexes of thiamine with $Cd^{2+}$, $Zn^{2+}$, $Pt^{2+}$, $Mn^{2+}$ etc containing mainly a M-$N_{1'}$ direct bonding were prepared in recent years and their structures were solved with X-rays [4].

The use however of the "active aldehyde" intermediates of thiamine as ligands for the formation of complexes with bivalent metal ions, presented the advantage of the delocalization of the net positive charge on $N_3$ of thethiazolium moiety to the sulfur atom and resulted to the easier formation of compounds with M-$N_{1'}$ bonds [3-7].

We summarize here all the techniques used thus far for the characterization of these complexes, adding a few more, as well as the conclusions drawn concerning the enzymatic action of thiamine.

Materials and Methods: The preparation of the ligands (L) 2-(α-hydroxybenzyl)thiamine = HBT and 2-(α-hydroxycyclohexyl-methyl) thiamine = HCMT and the complexes $MLCl_3$ ($M=Zn^{2+}$, $Cd^{2+}$, $Hg^{2+}$, $Co^{2+}$, $Ni^{2+}$) was described previously [3,6]. The structure of the complex Zn(2-(α-hydroxycyclohexylmethyl) thiamine)$Cl_3$ was solved with the same method and instrument used for the Hg(HBT)$Cl_3$ complex [3]. Crystals of the complex were grown by slowly diffusing acetone and ether in a solution of the complex in methanol. They were unstable in the absence of the solvents and were mounted in a sealed capillary and the data collection followed.

The 1D-$^1H$ nmr spectra were recorded on a Brucker AC 200 Spectrometer with TMS as an internal standard.

The 2D-$^1H$ nmr spectra were also obtained on a Brucker AC 200 spectrometer at 200 MHz. The pulse sequence $90^o$-$t_1$-$90^o$-$r_m$-$90^o$ was followed with mixing time of 500 ms.

The DRS and the solution UV-Vis spectra were recorded as described [6].

Results and Discussion: Metal complexes of the $II_B$ group metal ions as well as $Co^{2+}$, $Ni^{2+}$ and $Cu^{2+}$ were used for the preparation of the 1:1 complexes with the "active aldehyde" derivatives of the thiamin HBT and HCMT that corresponded to the general formulae $MLCl_3$, with L the above thiamine derivatives and M all the metal ions except $Cu^{2+}$ [3-7]. The latter was oxydizing both ligands to thiochrome [6,8], producing the complexes $[Cu^{II}L'Cl_2].H_2O$ and $[Cu^I(HL')Cl]^+[Cu^{II}Cl_3(MeOH)]^-$ [6]. The various techniques used were :

(i) X-ray crystal structures

The first crystal structure reported was the one of $Hg^{2+}$ with 2-(α-hydroxybenzyl)thiamine, Hg(HBT)$Cl_3$ [3]. Here the ligand had the less common S conformation with $\Phi_P$ = [N(3)- C(3,5') - C(5') - C(4') ] = 172.7° and $\Phi_T$ = [C(5')-C(3,5')-N(3)-C(2)]=-100.0° (Pletcher and Sax [9] defined the S conformation of thiamine with $\Phi_T=\pm100^o$, $\Phi_P=\pm150^o$, the V conformation with $\Phi_T=\pm90^o$, $\Phi_P=\pm90^o$ and the F conformation with $\Phi_T=0^o$, $\Phi_P=\pm90^o$). These was a direct Hg-$N_{1'}$ bonding of 2.23Å [3].

A second crystal structure of the compound Zn(2-(α-hydroxyclohexylmethyl)thiamine)$Cl_3$, Zn(HCMT)$Cl_3$ was subsequently solved but only to an R value of about 13.8%. Despite the low accurancy in the details of the structure, it is clear that the bonding of the metal takes place again with the $N_{1'}$ of the pyrimidine moiety (The bond distance Zn-$N_{1'}$ is about 2.1 Å).

The HCMT ligand is also in the S conformation with $\Phi_P \sim 172.8^o$ and $\Phi_T \sim -92.7^o$ and the configuration around Zn is pseudotetrahedral [3].

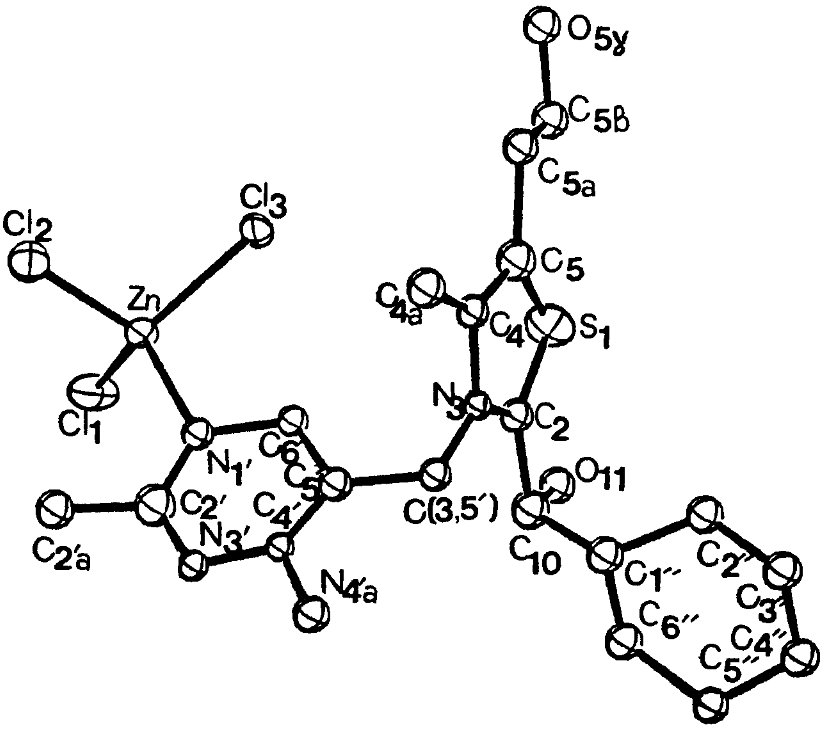

Fig.1. The structure of the complex $Zn(HCMT)Cl_3$

(ii) Vibrational IR-Raman Spectra

The comparison of the IR and Raman spectra of the various complexes with the ones of known structure, showed that they were all similar band by band, except the metal-ligand (M-N, M-Cl) streching vibrations [5]. Another difference of the various complexes was the difference at the position of the first νC=N vibration of the pyrimidine moiety, varying with the bulkiness of the $N_{1'}$ coordinated metal ion and the protonation. This frequency was increasing in the order $H^+ > Co^{2+} \sim Ni^{2+} \sim Zn^{2+} > Cd^{2+} > Hg^{2+}$ [3,5].

(iii) NMR Spectra

Comparison of the $^{13}C$ nmr of the various metal complexes in solution and the solid state showed that they were all isostructural in both phases, to the ones with known structures and that both the M-$N_{1'}$ bonding and the S conformation of the ligands were retained in $D_2O$ and DMSO-$d_6$ solutions [7]. This was also confirmed with the $^{199}Hg$ nmr spectra [7] of the mercury complexes.

(a) $^1H$ nmr spectra (1D): The $^1H$ nmr chemical shifts of the various ligands and their $II_B$ metal complexes in DMSO-$d_6$ solutions, are included in Table I.

Protonation of both ligands at $N_{1'}$ of pyrimidine causes downfield shifts to its adjacent protons, $C_{6'}$-H and $C_{2'}$-$CH_3$. This however, does not cause significant changes to the neighboring coupling constants (Table I), indicating the absence of conformational changes of the molecule upon protonation.

The broad bands of HBT.HCl and HCMT.HCl located at ~8.9 and 9.254 ppm respectively, are assigned to the N(4'a)$H_2$ protons in exchange with their N(1')-H protons. This is evidenced from the fact that in the non protonated ligands, they are located at 7.3-7.5 ppm as sharp bands as expected for aminopyrimidines [10].

Unexpectedly, the neighboring protons to the $N_{1'}$ site of pyrimidine are not shifted significantly upon metal coordination.

In a first approach this can be explained as the result of two opposite effects : (a) The localization of the net negative charge of the $[MCl_3]^-$ ion at the $N_{1'}$ position of the pyrimidine, causing an upfield shift of the adjacent protons and (b) the metal complexation at $N_{1'}$ causing an opposite downfield shift of the same protons [11,12]. Similar were the results of the $^{13}C$ nmr in $D_2O$ and DMSO-$d_6$ solutions for the carbon atoms adjacent to $N_{1'}$ that can not be due to the breaking of the M-$N_{1'}$ bonding in solution, since the behavior was also similar in the solid state $^{13}C$ nmr spectra [7].

All the protons of pyrimidine in HBT, HBT.HCl and their complexes are more deshielded compared to the ones of HCMT, HCMT.HCl and their complexes and therefore more upfield shifted than the latter (Table I). This is due to the stacking effect of the benzyl ring, being almost parallel to the pyrimidine in the HBT derivatives (dihedral angle $9^o$ and shorter distance 3.4Å) [3,13,14]. The same was observed in the solution and solid state $^{13}C$ nmr spectra of all the HBT and HCMT complexes for the adjacent to $N_{1'}$ carbon atoms [7].

The O(5γ)H, C(3,5')$H_2$, C(10)-H and O(11)-H protons on the other hand, were more influenced upon complexation than protonation. They were all upfield shifted with the same magnitude for the complexes of both ligands with the three metals.

These results may be explained by the facts : (a) that the methylenic C(3,5')$H_2$ protons and the C(10)H proton are in close contact (electrostatic interaction) with

Table I. $^1H$ nmr spectral data of the compounds

| Compound | O(11)-H | N(4α')H | C(6')-H | C(10)-H | C(3,5')$H_2$ | O(5γ)H | C(5β)$H_2$ | C(5α)$H_2$ | 2'-$CH_3$ | 4-$CH_3$ |
|---|---|---|---|---|---|---|---|---|---|---|
| HBT | 8.012<br>$^3J_{11,10}$=4.86 | 7.280 | 6.851 | 6.590<br>$^3J_{10,11}$=4.87 | 5.782, 5.526<br>$^2J_{AB}$=17.00 | 5.514<br>$^3J_{\gamma,\beta}$=5.09 | 3.787 | 3.152<br>$^3J_{\alpha,\beta}$=5.33 | 2.361 | 2.351 |
| HBT.HCl | 7.973<br>$^3J_{11,10}$=4.77<br>(-0.039) | ~8.9ᵃ | 7.232<br>(0.381) | 6.542<br>$^3J_{10,11}$=4.71<br>(-0.048) | 5.765, 5.542<br>$^2J_{AB}$=18.03<br>(-0.017),(0.016) | ~5.5ᵃ | 3.816<br>(0.029) | 3.165<br>$^3J_{\alpha,\beta}$=5.51<br>(0.013) | 2.559<br>(0.198) | 2.415<br>(0.064) |
| Zn(HBT)$Cl_3$ | 7.775<br>$^3J_{11,10}$=4.60<br>(-0.237) | 7.219<br>(-0.061) | 6.909<br>(0.058) | 6.481<br>$^3J_{10,11}$=4.60<br>(-0.109) | 5.441<br>-0.341),(-0.083) | 5.416<br>$^3J_{\gamma,\beta}$=4.96<br>(-0.098) | 3.814<br>(0.027) | 3.160<br>$^3J_{\alpha,\beta}$=5.44<br>(0.008) | 2.403<br>(0.042) | 2.358<br>(0.007) |
| Cd(HBT)$Cl_3$ | 7.754<br>$^3J_{11,10}$=4.62<br>(-0.258) | 7.186<br>(-0.094) | 6.850<br>(~0) | 6.483<br>$^3J_{10,11}$=4.62<br>(-0.107) | 5.443<br>(-0.339),(-0.083) | 5.406<br>$^3J_{\gamma,\beta}$=5.00<br>(-0.108) | 3.813<br>(0.026) | 3.160<br>$^3J_{\alpha,\beta}$=5.40<br>(0.008) | 2.370<br>(0.009) | 2.354<br>(0.003) |
| Hg(HBT)$Cl_3$ | 7.752<br>$^3J_{11,10}$=4.57<br>(-0.260) | 7.211<br>(-0.069) | 6.862<br>(0.011) | 6.476<br>$^3J_{10,11}$=4.55<br>(-0.114) | 5.435<br>(-0.347),(-0.091) | 5.409<br>$^3J_{\gamma,\beta}$=4.91<br>(-0.105) | 3.813<br>(0.026) | 3.158<br>$^3J_{\alpha,\beta}$=5.38<br>(0.006) | 2.382<br>(0.021) | 2.353<br>(0.002) |
| HCMT | 7.385<br>$^3J_{11,10}$=5.12 | 7.501 | 7.348 | 5.264<br>$^3J_{10,11}$=5.10 | 5.837, 5.609<br>$^2J_{AB}$=17.10 | 5.490<br>a | 3.763 | 3.147<br>a | 2.399 | 2.433 |
| HCMT.HCl | 7.398<br>a<br>(0.013) | 9.254<br>(1.753) | 7.791<br>(0.443) | 5.254<br>a<br>(-0.010) | 5.897, 5.687<br>$^2J_{AB}$=17.90<br>(0.060),(0.078) | ~5.5ᵃ | 3.788<br>(0.025) | 3.144<br>$^3J_{\alpha,\beta}$=5.41<br>(~0) | 2.645<br>(0.246) | 2.427<br>(-0.006) |
| Zn(HCMT)$Cl_3$ | 7.141<br>$^3J_{11,10}$=5.12<br>(-0.244) | 7.316<br>(-0.185) | 7.316<br>(-0.032) | 5.182<br>$^3J_{10,11}$=5.10<br>(-0.082) | 5.570<br>(-0.280),(-0.052) | 5.382<br>$^3J_{\gamma,\beta}$=4.95<br>(-0.108) | 3.796<br>(0.033) | 3.138<br>$^3J_{\alpha,\beta}$=5.39<br>(-0.009) | 2.468<br>(0.069) | 2.399<br>(-0.034) |
| Cd(HCMT)$Cl_3$ | 7.122<br>$^3J_{11,10}$=5.10<br>(-0.263) | 7.288<br>(-0.213) | 7.288<br>(-0.060) | 5.179<br>$^3J_{10,11}$=5.10<br>(-0.085) | 5.550<br>(-0.287),(-0.059) | 5.372<br>$^3J_{\gamma,\beta}$=4.90<br>(-0.118) | 3.797<br>(0.034) | 3.138<br>$^3J_{\alpha,\beta}$=5.38<br>(-0.009) | 2.456<br>(0.057) | 2.397<br>(-0.036) |
| Hg(HCMT)$Cl_3$ | 7.120<br>$^3J_{11,10}$=5.15<br>(-0.265) | 7.327<br>(-0.174) | 7.301<br>(-0.047) | 5.173<br>$^3J_{10,11}$=5.12<br>(-0.091) | 5.546<br>(-0.291),(-0.063) | 5.380<br>$^3J_{\gamma,\beta}$=4.94<br>(-0.110) | 3.801<br>(0.038) | 3.139<br>$^3J_{\alpha,\beta}$=5.42<br>(-0.008) | 2.466<br>(0.067) | 2.401<br>(-0.032) |

all three chloride ions of $Hg^{2+}$ (~3.5Å) as found in the crystal structure of $Hg(HBT)Cl_3$ [3]. The same is true in the structure of $Zn(HCMT)Cl_3$ that does not change in solution as well [7]. It should be noted here that the $C(3,5')H_2$ methylenic protons behave as an AB system in the protonated and non protonated ligands due to the chiral center at C(10) [15]. In their metal complexes however, they are seen as isochronous. To account for the observed upfield shift of the O(5γ)H proton, we compare the torsional angles $\Phi_{5\alpha}$ and $\Phi_{5\beta}$ of the complexes $Hg(HBT)Cl_3$ and the ligand HBT.HCl.

Thus in the complex $Hg(HBT)Cl_3$ with $\Phi_{5\alpha}=78.6^o$ and $\Phi_{5\beta}=65.5^o$, the thiazolium side chain is brought under the influence of the aromatic thiazolium ring itself. This causes the upfield shift of the O(5γ)H proton, being under the influence of the ring. In the ligand HBT.HCl on the other hand, the thiazolium side chain ($\Phi_{5\alpha}=3.3^o$, $\Phi_{5\beta}=63.4^o$) is directed far away and not influenced at all by the aromatic thiazolium ring.

<u>(b) $^1$H nmr spectra (2D, NOESY)</u>: In order to detect whether or not a stacking

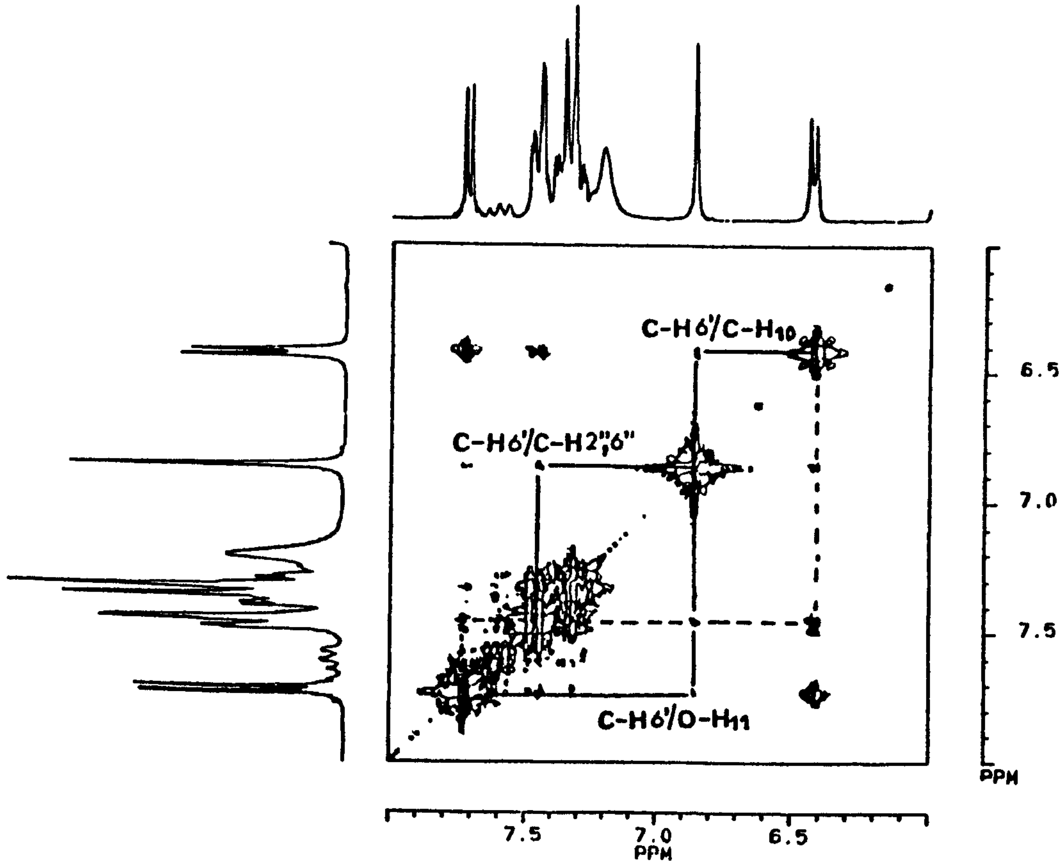

Fig.2. $^1$H nmr NOESY spectrum (contour plot) of the complex $Zn(HBT)Cl_3$, in DMSO-$d_6$.

interaction between the benzyl and the pyrimidine rings found in the solid state [3] persists also in solution, we have recorded the 2D-NOE spectrum of the complex $Zn(HBT)Cl_3$ in DMSO-$d_6$ (Fig.2). For comparison the 2D-NOE spectrum of the complex $Zn(HCMT)Cl_3$ was recorded as well. From Fig.2, it is easily seen that enhanced connectivities exist between the protons C(6')-H/C(10)-H, C(6')-H/C(2",6")-H and a weak cross peak between C(6')-H and O(11)-H. Therefore the pyrimidine C(6')-H proton approaches the C(10)-H and O(11)-H and the C(2",6") protons of the benzene ring. This indicates that the benzene and pyrimidine rings are almost parallel, like in the solid phase.

Comparison of the 2D-NOE spectra of the complexes $Zn(HBT)Cl_3$ and $Zn(HCMT)Cl_3$ show that in the second case there were not observed any cross peak connectivities between pyrimidine and cyclohexane, thus excluding any interaction (stacking, hydrophobic) between these two rings, as this is also true in the solid phase.

(iv) Electronic Spectra

The $Co^{2+}$ and $Ni^{2+}$ complexes of the general formulae $MLCl_3$ should have similar structures ($M\text{-}N_{1'}$ bonding and S conformation of the ligands, pseudotetrahedral structures around the metals) with the ones of known structures $Hg(HBT)Cl_3$ and $Zn(HCMT)Cl_3$ as exposed above (similar IR-Raman spectra band by band). Their pseudotetrahedral structures are further substantiated by their electronic DRS (diffuse reflectance spectra) and DMF solution spectra (Table II). In the DRS of the $CoLCl_3$ complexes the multiple bands near 6600 $cm^{-1}$ and 15700 $cm^{-1}$ correspond to the $^4A_2(F) \rightarrow {}^4T_1(F)$ and $^4A2(F) \rightarrow {}^4T_1(P)$ transitions respectively, in a tetrahedral environment [16]. The multiplicity of the bands however show reduction of $T_d$ to $C_{3v}$ symmetry (trigonal distortion). The various components of the multiple bands are assigned as follows : the one near 5200 $cm^{-1}$ to $^4A_2(F) \rightarrow {}^4A_2(T_1,F)$, the ones near 6600 and 7400 $cm^{-1}$ to the $^4A_2(F) \rightarrow {}^4E(T_1,F)$, the 14800 $cm^{-1}$ to the $^4A_2(F) \rightarrow {}^4A_2(T_1,P)$ and the 15700 and 16500 $cm^{-1}$ to the $^4A_2(F) \rightarrow {}^4E(T_1,P)$ transition [17-19]. Since two bands correspond to each of the $^4A_2(F) \rightarrow {}^4E(T_1,F)$ and $^4A_2(F) \rightarrow {}^4E(T_1,P)$ transitions, the $^4E$ term might further split to $B_1$ and $B_2$ levels.

The same geometries are retained in DMF solutions, while in $D_2O$ they are transformed to octahedral ones (Table II) and the band observed near 20000 $cm^{-1}$ is assigned to the $^4T_{1g} \rightarrow {}^4T_{1g}(P)$ transition [16].

| Table II. Vis and near IR spectral data of the compounds | | | | |
|---|---|---|---|---|
| Compound | Maxima (kK) | Assignments in $T_d$ symmetry | Assignments in $C_{3v}$ symmetry | Solvent |
| Co(HBT)$Cl_3$<br>10Dq=3722$cm^{-1}$<br>B=734$cm^{-1}$ | 5.21<br>6.69<br>7.44<br>14.99<br>15.72<br>16.47 | $^4A_2(F) \rightarrow {}^4T_1(F)$<br>$^4A_2(F) \rightarrow {}^4T_1(P)$ | $^4A_2(F) \rightarrow {}^4A_2(T_1,F)$<br>$^4A_2(F) \rightarrow {}^4E(T_1,F)$<br>$^4A_2(F) \rightarrow {}^4A_2(T_1,P)$<br>$^4A_2(F) \rightarrow {}^4E(T_1,P)$ | DRS |
| Co(HBT)$Cl_3$<br>10Dq=4026$cm^{-1}$<br>B=719$cm^{-1}$ | 6.49<br>6.94<br>7.40<br>14.88<br>16.44 | $^4A_2(F) \rightarrow {}^4T_1(F)$<br>$^4A_2(F) \rightarrow {}^4T_1(P)$ | $^4A_2(F) \rightarrow {}^4A_2(T_1,F)$<br>$^4A_2(F) \rightarrow {}^4E(T_1,F)$<br>$^4A_2(F) \rightarrow {}^4A_2(T_1,P)$<br>$^4A_2(F) \rightarrow {}^4E(T_1,P)$ | DMF |
| Co(HBT)$Cl_3$ | 19.68<br>21.27 | $4T_{1g} \rightarrow 4T_{1g}(P)$ | in octahedral environment | $H_2O$ |
| Co(HCMT)$Cl_3$<br>10Dq=3665$cm^{-1}$<br>B=734$cm^{-1}$ | 5.14<br>6.55<br>7.40<br>14.71<br>15.69<br>16.53 | $^4A_2(F) \rightarrow {}^4T_1(F)$<br>$^4A_2(F) \rightarrow {}^4T_1(P)$ | $^4A_2(F) \rightarrow {}^4A_2(T_1,F)$<br>$^4A_2(F) \rightarrow {}^4E(T_1,F)$<br>$^4A_2(F) \rightarrow {}^4A_2(T_1,P)$<br>$^4A_2(F) \rightarrow {}^4E(T_1,P)$ | DRS |
| Co(HCMT)$Cl_3$<br>10Dq=4028$cm^{-1}$<br>B=716$cm^{-1}$ | 6.48<br>6.98<br>7.39<br>14.78<br>16.42 | $^4A_2(F) \rightarrow {}^4T_1(F)$<br>$^4A_2(F) \rightarrow {}^4T_1(P)$ | $^4A_2(F) \rightarrow {}^4A_2(T_1,F)$<br>$^4A_2(F) \rightarrow {}^4E(T_1,F)$<br>$^4A_2(F) \rightarrow {}^4A_2(T_1,P)$<br>$^4A_2(F) \rightarrow {}^4E(T_1,P)$ | DMF |
| Co(HCMT)$Cl_3$ | 19.82<br>21.13 | $^4T_{1g} \rightarrow {}^4T_{1g}(P)$ | in octahedral environment | $H_2O$ |
| Ni(HBT)$Cl_3$<br>10Dq=4704$cm^{-1}$<br>B=852$cm^{-1}$ | 4.39<br>5.12<br>8.69<br>15.04<br>15.87<br>17.24 | $^3T_2(F) \rightarrow {}^3A_2(F)$<br>$^3T_1(F) \rightarrow {}^3A_2(F)$<br>$^3T_1(F) \rightarrow {}^3T_1(P)$ | $^3E(T_1,F) \rightarrow {}^3E(T_2,F)$<br>$^3E(T_1,F) \rightarrow {}^3A_2(F)$<br>$^3E(T_1,F) \rightarrow {}^3E(T_1,P)$<br>$^3E(T_1,F) \rightarrow {}^3A_2(T_1,P)$ | DRS |
| Ni(HBT)$Cl_3$<br>10Dq=4390$cm^{-1}$<br>B=860$cm^{-1}$ | 8.29<br>14.55<br>16.13<br>17.35 | $^3T_1(F) \rightarrow {}^3A_2(F)$<br>$^3T_1(F) \rightarrow {}^3T_1(P)$ | $^3E(T_1,F) \rightarrow {}^3A_2(F)$<br>$^3E(T_1,F) \rightarrow {}^3E(T_1,P)$<br>$^3E(T_1,F) \rightarrow {}^3A_2(T_1,P)$ | DMF |
| Ni(HBT)$Cl_3$ | 13.96<br>25.72 | $^3A_{2g} \rightarrow {}^3T_{1g}$<br>$^3A_{2g} \rightarrow {}^3T_{1g}(P)$ | in octahedral environment | $H_2O$ |

The crystal field strength 10Dq was about 3700 $cm^{-1}$ in the solid state and about 4000 $cm^{-1}$ in DMF solutions. The approximately equal value of the Racah parameter B for both $Co^{2+}$ complexes on the other hand, indicate a comparable covalent character in their Co-$N_{1'}$ bonds.

In the case of the Ni(HBT)$Cl_3$ the multiple bands near 8700 $cm^{-1}$ and 15900

$cm^{-1}$ in the DRS are due to the $^3T_1(F) \longrightarrow {}^3A_2(F)$ and $^3T_1(F) \longrightarrow {}^3T_1(P)$ transitions in a tetrahedral environment [16]. Their multiplicity is again due to a trigonal distortion ($C_{3V}$ symmetry) (See Table II) [18]. In DMF solutions the geometry is retained but it becomes again octahedral in aqueous solutions (See Table II). The band at 4390 $cm^{-1}$ is assigned to the ($v_1$) $^3T_2(F) \longrightarrow {}^3A_2(F)$ transition. Therefore $10Dq=v_1=4390$ $cm^{-1}$.

Finally, the ligand HBT shows a maximum at 274 nm, the HBT.HCl at 276 nm and the complexes $Co(HBT)Cl_3$ and $Ni(HBT)Cl_3$ at 275.5 and 275 nm respectively, in DMF solutions . The HCMT, HCMT.HCl and $Co(HCMT)Cl_3$ show the same band at 272, 273 and 272.5 nm correspondingly. This band is assigned to a $\pi \longrightarrow \pi^*$ transition of the pyrimidine ring of thiamine. Its bathochromic shift in the protonated and metallated ligands indicate the involvement of the pyrimidine ($N_{1'}$) moiety to protonation and metallation [20].

Concluding Remarks: The easy formation of metal complexes with the "active aldehyde" derivatives of thiamine with a $M-N_{1'}$ bonding, may indicate that the intervention of the metal ions follows the formation of these intermediates during the enzymatic process. The S conformation of the ligand seems to be important during the enzymatic action, since it persists in all the C(2a) substituted thiamine derivatives. (The V conformation of thiamine and the pyrophosphate metal binding are also important, after liberation of the C(2a) substituent, since it was recently found in the structure of transketolase, containing $Ca^{2+}$ ions [21].

References:

[1] N.Hadjiliadis and J.Markopoulos, Chim.Chron.(New Series), 10, 1 (1981).

[2] R.Breslow and E.McNelis, J.Am.Chem.Soc., 81, 3080 (1959).

[3] M.Louloudi, N.Hadjiliadis, J.A.Feng, S.Sukumar and R.Bau, J.Am.Chem.Soc., 112, 7233 (1990) and references there in.

[4] M.Louloudi and N.Hadjiliadis, Coord.Chem.Revs., submitted for publication.

[5] N.Hadjiliadis, M.Louloudi and I.S.Butler, Spectrochim.Acta, 47A, 445 (1991).

[6] M.Louloudi and N.Hadjiliadis, J.Chem.Soc.,Dalton, 1635 (1991)

[7] M.Louloudi, N.Hadjiliadis and I.S.Butler, J.Chem.Soc., Dalton, 1401 (1992).

[8] M.Louloudi, N.Hadjiliadis and I.S.Butler, Inorg.Chim.Acta, submitted for publication.

[9] J.Pletcher and M.Sax, J.Am.Chem.Soc., 94, 3998 (1972).

[10] N.J.Oppenheimer, L.O.Rodriguez and S.M.Hecht, Biochemistry, 19, 4096 (1980).

[11] R.H.Bible, "Interpretation of NMR spectra" Plenum Press, New York (1965).

[12] L.M.Jackmann and S.Sternhell, "Applications of NMR Spectroscopy in Organic Chemistry", 2nd Edition, Pergamon Press (1969).

[13] J.Pletcher, M.Sax, G.Blank and M.Wood, J.Am.Chem.Soc., 99, 1396 (1977).

[14] J.Mieyal, G.Bantle, R.Votaw, I.Rosner and H.Sable, J.Biol.CHem., 246, 5213 (1971).

[15] R.Kluger, Ann.N.Y.Acad.Sci., 378, 63 (1982).

[16] A.B.P.Lever, "Inorganic Electronic Spectroscopy", Elsevier Publishing Co. (1968).

[17] R.M.Gaura, P.Stein, R.D.Willett and D.X.West, Inorg.Chim. Acta, 60, 213 (1982).

[18] B.B.Garrett, V.L.Goedken and J.V.Quagliano, J.Am.Chem.Soc., 92, 489 (1970).

[19] G.Marcotrigiano, L.Menabue and G.C.Pellacani, Inorg.Chim. Acta, 26, 57 (1978).

[20] N.Hadjiliadis, P.Kourounakis and T.Theophanides, Inorg.Chim. Acta, 7, 226 (1973).

[21] Y.Lindqvist, G.Schneider, U.Ermler and M.Sundstrom, EMBO Journal, 11, 2373 (1992).

**Received: June 1, 1993**

